# Degradation of Hydrogels Based on Potassium and Sodium Polyacrylate by Ionic Interaction and Its Influence on Water

**DOI:** 10.3390/polym14132656

**Published:** 2022-06-29

**Authors:** Diego David Pinzon-Moreno, Isabel Rosali Maurate-Fernandez, Yury Flores-Valdeon, Antony Alexander Neciosup-Puican, María Verónica Carranza-Oropeza

**Affiliations:** 1 Faculty of Chemistry and Chemical Engineering, National University of San Marcos, Lima 15081, Peru; isabel.maurate@unmsm.edu.pe (I.R.M.-F.); yfvaldeon071@gmail.com (Y.F.-V.); mcarranzao@unmsm.edu.pe (M.V.C.-O.); 2 Centro de Investigaciones Tecnológicas, Biomédicas y Medioambientales, National University of San Marcos, Lima 15081, Peru; antony.neciosup@unmsm.edu.pe

**Keywords:** hydrogel, polymer degradation, potassium and sodium polyacrylate, swelling, physicochemical changes in the water, polymeric nanoparticles

## Abstract

Hydrogels are a very useful type of polymeric material in several economic sectors, acquiring great importance due to their potential applications; however, this type of material, similarly to all polymers, is susceptible to degradation, which must be studied to improve its use. In this sense, the present work shows the degradation phenomena of commercial hydrogels based on potassium and sodium polyacrylate caused by the intrinsic content of different types of potable waters and aqueous solutions. In this way, a methodology for the analysis of this type of phenomenon is presented, facilitating the understanding of this type of degradation phenomenon. In this context, the hydrogels were characterized through swelling and FTIR to verify their performance and their structural changes. Likewise, the waters and wastewaters used for the swelling process were characterized by turbidity, pH, hardness, metals, total dissolved solids, electrical conductivity, DLS, Z-potential, and UV-vis to determine the changes generated in the types of waters caused by polymeric degradation and which are the most relevant variables in the degradation of the studied materials. The results obtained suggest a polymeric degradation reducing the swelling capacity and the useful life of the hydrogel; in addition, significant physicochemical changes such as the emergence of polymeric nanoparticles are observed in some types of analyzed waters.

## 1. Introduction

Currently, the use of hydrogels has gained popularity because of their intrinsic property of retaining significant amounts of water or other fluids, being increasingly used in various sectors, and becoming a material that presents itself as a strategic alternative to many current problems associated with consumption, management, conservation, optimization, and release of water and/or aqueous solutions of interest [1]. Consequently, hydrogels have gained space in several sectors such as agriculture, chemistry, electricity, electronics and magnetism, energy, environment and purification, mechanics, medicine-pharmacy, etc. [2,3,4,5,6,7,8,9,10,11,12], presenting themselves as a versatile and economical solution to facilitate the realization of different processes [1,13,14,15].

Hydrogels have a complex tangled three-dimensional structure that will traditionally consist of a polymer chain, polar side groups, and crosslinkings; these fractions of the polymeric network can absorb, swell, retain and release controlled amounts of water, liquids, or aqueous solutions [13]. The previously commented parts that constitute the hydrogel provide important characteristics to this type of material; in the case of the polymeric chain, it supports other elements such as polar side groups and multiple crosslinkings, as well as providing flexibility to the polymer and allowing their dilation. Polar lateral groups provide hydrophilic characteristics to hydrogels providing water affinity. On the other hand, crosslinkings, which are bridges that connect the polymer chains with the help of molecules called crosslinkers that contain two or more reactive points capable of attaching to specific functional groups [16,17]. Thus, it creates a network and consequently a porous material that allows for the storage of significant amounts of water or other fluids. Naturally, this set of elements enables the functionality of hydrogels by determining the mass flows of absorption, releasing further the storage and retention capacity of liquids that this material can have during their useful life. However, this ability allows several agents to penetrate the polymeric network during the swelling process, directly interacting; this can have negative effects on the structure, decreasing the efficiency and useful life of this type of polymer [15,18].

In this context, the stability of this type of material must be studied to understand its behavior, efficiency, and useful life when subjected to different conditions. The different types of degradation existing in polymers (mechanical, chemical, photo-oxidative, catalytic, thermal, biological, etc.) [19,20,21,22] suggest that, when faced with different types of stimuli, hydrogels can undergo changes that alter their properties, as well as altering aqueous media in contact with this type of polymer [23,24]. The absorption-release mass fluxes of a hydrogel depend on different environmental factors and are also determined by the interactions between the polymeric and aqueous phases. In this way, substrates can be dragged from one phase to another, a phenomenon induced by the different types of degradation, logically generating cross-contamination that should be considered as an analysis criterion depending on the complexity of the hydrogel application [25]. Examples of this type of care can be found in pharmaceutical applications during the release of substrates in the body or in the case of agriculture in the release of substrates in the soil. Therefore, the composition of hydrogels should guarantee that the surrounding environment is not contaminated or harmed by any type of residue. Alternatives to avoid such contamination are through the stabilization of this type of polymer or the reduction in toxic reagents as base materials of hydrogels.

Consequently, several authors have shown that the efficiency concerning swelling can vary depending on the type of salts, solvents, solids, colloids, aggregates, minerals, chemicals, etc. that integrate into the water and that swell the hydrogel [26]; even so, the ionic exchange can change the polymer and the surrounding environment [13,18]. In this context, these agents can interact during the swelling process with different parts of the hydrogel’s three-dimensional structural network (polymer chain, lateral polar functional groups, crosslinkings, etc.) modifying it partially or totally during service [15,18,23]. This particularity of hydrogels must be characterized to optimize their use, to know their useful life, properties physical and chemical, degradation phenomena, release of substrates absorbed, swelling ratio, among other important aspects [13,27].

In this context, this work analyzed two types of hydrogels based on potassium and sodium polyacrylate exposed to different types of commercial waters and aqueous solutions based on NaCl, KCl, CaCl_2_•2H_2_O, MgCl_2_•6H_2_O, etc. A progressive degradation behavior was verified, changing the structure of the hydrogel and changing the physicochemical composition of the waters and the prepared aqueous solutions that interacted with the hydrogel. Furthermore, the hydrogel was characterized by gravimetric swelling experiments in different types of waters and solutions, and to being analyzed by FTIR before and after swelling characterization. On the other hand, the different types of waters and solutions were analyzed through physical-chemical analysis, DLS, Z-potential, UV-vis, metal content, turbidity, hardness, pH, conductivity, etc. The results obtained demonstrate the polymeric degradation of hydrogels, leading to a reduction in their respective performance in terms of swelling, retention, and useful life, and the physicochemical alteration of the different waters and prepared solutions. Finally, a methodology to verify and establish this type of polymeric degradation in hydrogels is presented.

## 2. Materials and Methods

### 2.1. Materials

Two types of commercial polyacrylate hydrogels based on potassium and sodium, synthesized by polymerization in aqueous solution, were purchased with characteristics recorded in Table 1 and Table 2. The references of all analyzed samples can be consulted in Table 3. Different types of commercial waters were used for the swelling process; their physicochemical characteristics can be seen in Table 4. Moreover, sodium Chloride NaCl, Potassium Chloride KCl, and Calcium Chloride CaCl_2_•2H_2_O (MW: 56.11, 74.55, and 147.01, respectively) from Central Drug House and Magnesium Chloride MgCl_2_•6H_2_O (MW: 203.30) from Himedia were used to prepare the saline solutions. Additionally, Calcium Ca, Calcium Nitrate tetrahydrate Ca(NO_3_)_2_•4H_2_O, and Calcium Carbonate CaCO_3_ (MW: 100.08) from Sigma-Aldrich were used to generate calcium-based solutions. All of the solutions were prepared by mechanical stirring at room temperature, keeping the indicated concentrations of Table 4. Solutions based on salts and solutions based on calcium were prepared with contents indicated in the physicochemical characterization of the types of waters, see Table 3 and Table 4.

### 2.2. Methods and Characterizations

To verify phenomena associated with hydrogel degradation or transformations due to exposure to different types of waters, the hydrogels and waters were characterized before and after the swelling process. Thus, hydrogels based on potassium and sodium polyacrylate were tested for swelling by the tea bag methodology employing three types of commercial waters (see Table 3). For all of the swelling tests, approximately 0.05 g of hydrogel per 100 mL of water was used; also, the swelling tests were performed in duplicate. Hydrogels were tested for swelling with distilled and osmotic water as references. Additionally, hydrogels were characterized by FTIR (Shimadzu IRTracer-100 spectrometer with Pike MIRacle single reflection horizontal ATR accessory) before and after the swelling process.

On the other hand, the characteristics of turbidity were carried out using an EPA Compliant Turbidity and Free & Total Chlorine Meter–Hanna Instruments S.L. HI-93414-02 (SMEWW-APHA-AWWA-WEF Part 2130B.22nd Ed. methodology); total water hardness was determined using a Sartorius SE2 Ultra-micro balance (SMEWW-APHA-AWWA-WEF Part 2340-C.23nd Ed. methodology); total metals were carried out using a PerkinElmer Optima 4300 DV ICP-OES Spectrometer (EPA method 200.7); total dissolved solids were determined using a Sartorius SE2 Ultra-micro balance (SMEWW-APHA-AWWA-WEF Part 2540C B.23 nd Ed. methodology); electrical conductivity was determined using a HI-9033 Heavy Duty Waterproof Portable Conductivity Meter; pH was determined using a HI-2020-01 edge^®^ Multiparameter pH Meter; Dynamic Light Scattering (DLS) by number-weighted and Zeta-potential by Phase Analysis Light Scattering and Frequency Analysis modes tests were performed on a NICOMP Nano Z3000 System; and UV-vis test performed on a Libra S22, Biochrom, Ltd., Cambridge, England. All of these tests were performed before and after the swelling process.

## 3. Results and Discussion

During the swelling test, several changes and transitions were visually observed in the hydrogels when submitted to commercial waters (WC and WSM) compared to distilled and/or osmotic waters. When commercial hydrogels (HK and HNa) are exposed to commercial waters (WC and WSM), there are changes such as a lower swelling, an inflection point showing a smaller maximum swelling compared to distilled water, and later, a loss of partial or total water retention capacity. On the other hand, when removing the bags (filter) that contain the commercial hydrogels from the commercial water baths, it is observed that the wastewater presents a significant change in turbidity. These visually appreciable changes are presented in-depth below using different characterization methods.

### 3.1. Swelling Test in Commercial Waters

**Figure 1:**
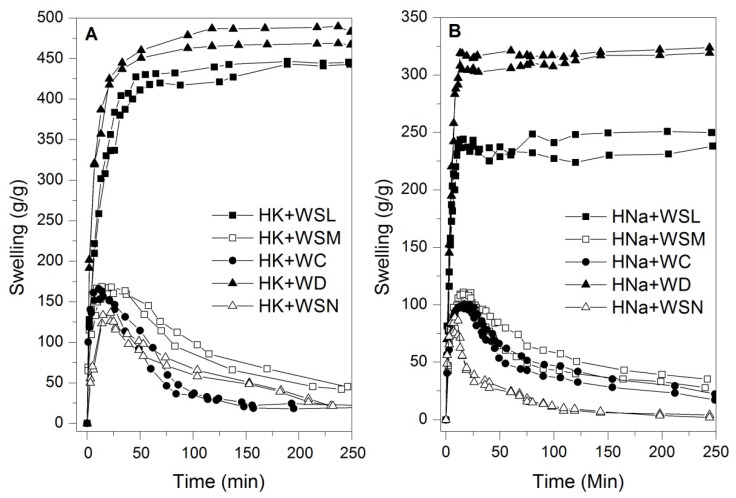
Swelling kinetics of hydrogels in types of waters. (**A**) HK and (**B**) HNa.

Figure 1 shows the kinetic swelling curves of commercial hydrogels based on potassium and sodium exposed to different types of waters. It is observed that, in general terms, potassium-based hydrogels perform better in terms of swelling than sodium-based hydrogels, regardless of the solution used for the test. In the same Figure, it is observed that distilled water allows for better performances concerning swelling, ~475 and ~320 g/g for HK and HNa, respectively, followed by the curves related to swelling with WSL in both types of hydrogels, around ~440 and ~250 g/g for HK and RNa, respectively; note that hydrogels exposed to the two types of water previously described (WD and WSL) reach equilibrium swelling. This difference in swelling between the two types of hydrogels may be associated with factors such as variations in polymer synthesis formulations, changes in the hydrogel particle size, as well as the electronegativity of the ions that make up the polar groups of the polymer chain of each hydrogel. Moreover, hydrogels exposed to the reference waters WC, WSM, and WSN present a phenomenon that significantly limits the swelling, transforming the kinetic curve, reaching inflection points that later decrease until to lose their character as superabsorbent materials without reaching an equilibrium swelling. These repeated trends suggest that WC, WSM, and WSN referenced waters contain substrates that limit swelling dramatically, distinct from WD and WSL referenced waters.

After these swelling tests, the hydrogels were dried on their respective filters at 40 °C until reaching a constant mass to be retested for swelling for a second time. In this new swelling test, it was observed that both types of hydrogels exposed to WD, and WSL water reached kinetic curves similar to the curves of the first swelling; however, hydrogels exposed to the reference waters WC, WSM, and WSN did not have responses that indicated swelling. This phenomenon could indicate structural changes in potassium and sodium hydrogels due to chemical agents present in the water, such as salts or other types of ions. In addition, the fact that it does not respond to second swelling attempts is one of the main indications of irreversible structural changes in the polymer, reducing the functionality of the hydrogels.

### 3.2. Swelling Test in Saline Solutions

**Figure 2:**
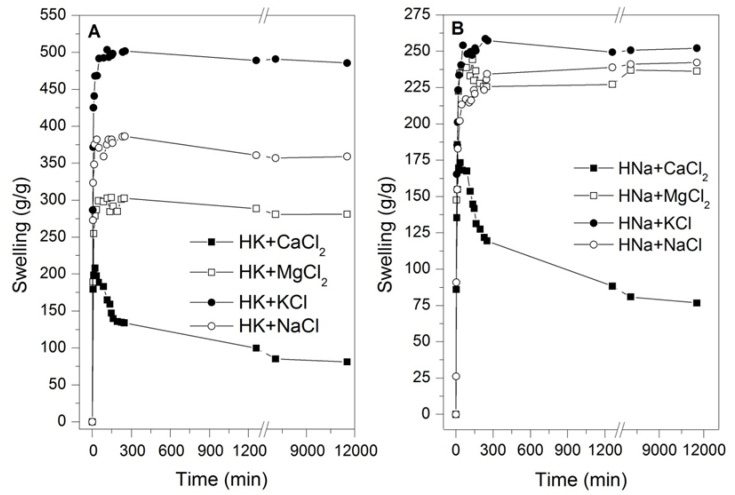
Swelling kinetics of hydrogels in saline solutions. (**A**) HK and (**B**) HNa.

In order to verify the swelling performance of hydrogels in different salts present in the different types of waters, different solutions were prepared in distilled water, maintaining the concentrations indicated in Table 4 related to calcium, magnesium, potassium, and sodium so that it was possible to determine the agent or agents that can generate the phenomenon associated with the partial or total reduction in the swelling capacity. In Figure 2, the kinetic curves of swelling of potassium and sodium-based hydrogels underexposure of the prepared solutions are shown. It should be noted that hydrogels show susceptibility depending on the type of solution, showing better swelling with potassium and sodium solutions, and lower performance with magnesium and calcium solutions. Lower swelling performances with magnesium and calcium solutions are visible, and it is observed that all of the curves reach equilibrium swelling except for the solutions prepared based on CaCl_2_, which present a swelling peak and later drop significantly after less than two hours of testing; this phenomenon was previously reported by other authors [28,29,30,31,32,33]. Furthermore, hydrogels show swellings that gradually decrease depending on the solution and show a kinetic curve with a function close to the WC, WSM, and WSN curves when exposed to CaCl_2_ (See Figure 2). Note that in the case of the sodium-based hydrogel, there is less susceptibility with slight variations with the MgCl_2_, KCl, and NaCl solutions but with a swelling considered as in equilibrium, which contrasts with a significant drop in the swelling curve with the CaCl_2_ solution.

### 3.3. Swelling Test in Calcium-Associated Solutions

**Figure 3:**
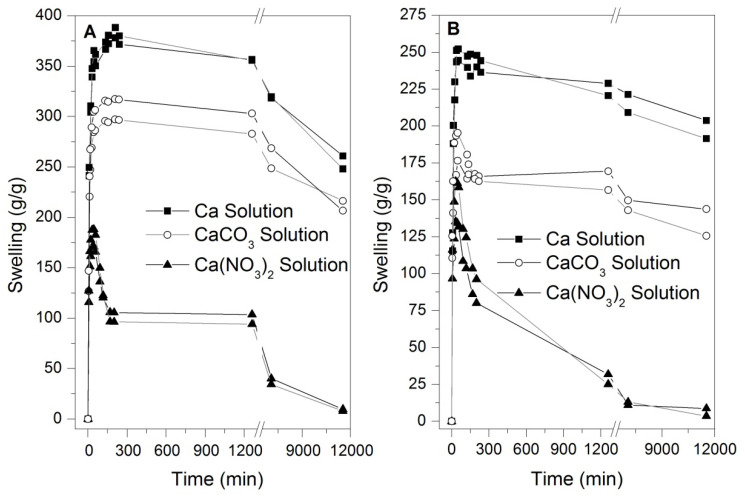
Swelling kinetics of hydrogels in calcium-associated solutions. (**A**) HK and (**B**) HNa.

Previous swelling results indicate that calcium-associated ions have the ability to limit and reduce the swelling capacity in potassium and sodium-based hydrogels over an exposure time. In this context, solutions were prepared based on Ca, CaCO_3_, and Ca(NO_3_)_2_ in distilled water, respecting the indicated concentration of calcium in Table 4. Figure 3 shows the kinetic swelling curves of commercial hydrogels based on potassium and sodium in calcium-associated solutions. In said curves, the same phenomenon described in Figure 1 and Figure 2 is observed in which the swelling reaches limited swelling peaks and a progressive drop in the solution’s retention capacity, in this context, it is observed that Ca, CaCO_3_, and Ca(NO_3_)_2_ cause inflection points and subsequently generate reductions in the retention capacity, proving to be more aggressive for the solution with Ca(NO_3_)_2_ reducing the superabsorbent capacity of hydrogels without reaching an equilibrium swelling.

The swelling curves presented in Figure 3 associated with calcium-based solutions, were formulated to contain the same amount of calcium in the water, presented variations in swelling between the hydrogels studied and the types of solutions, this behavior is due to the degree of ionization of these compounds in water, which in the case of Ca(NO_3_)_2_ presents complete ionization, allowing the ions of Ca^2+^ and NO_3_^−1^ to completely penetrate the structure of the hydrogels generating the degradation of the hydrogel by the ion exchange and crosslinking breakage, thus the swelling is reduced to zero in both hydrogels. In the case of CaCO_3_ and Ca, the ionization is partial and less significant compared to Ca(NO_3_)_2_, a condition that limits the number of ions generated in the solution and consequently their penetration into the polymeric networks of the hydrogel, therefore the swelling values are higher compared to the solution based on Ca(NO_3_)_2_ at all times of the test. The swelling tests associated with Figure 2 and Figure 3 suggest that the most reactive ions for the hydrogel network are Ca^2+^ and Mg^2+^, which significantly decrease the performance in terms of swelling and may reduce the entire swelling capacity in the case of Ca^2+^ ions. In this sense, cations such as Ca^2+^ are highly reactive, destabilizing the side groups (COO^−^) of the polymer chain; hence, this ionic alteration changes the polarity and hydrophilicity of the two types of hydrogels.

The negative effects produced by solutions in the presence of ions such as Ca^2+^ start immediately after the direct interaction between the polymeric and aqueous phases; this can be observed at the beginning of the kinetic curves that present a lower swelling/time rate. Degradation is also reflected in some kinetic curves of swelling that show points of infection or maximum swelling peaks that do not reach swellings comparable to those achieved with WD. Additionally, the drop in swelling without reaching equilibrium swelling shows a degradation of the polymer, which, added to the lack of response in second swelling cycles, ratifies structural changes in the hydrogels.

### 3.4. FTIR Analysis

**Figure 4:**
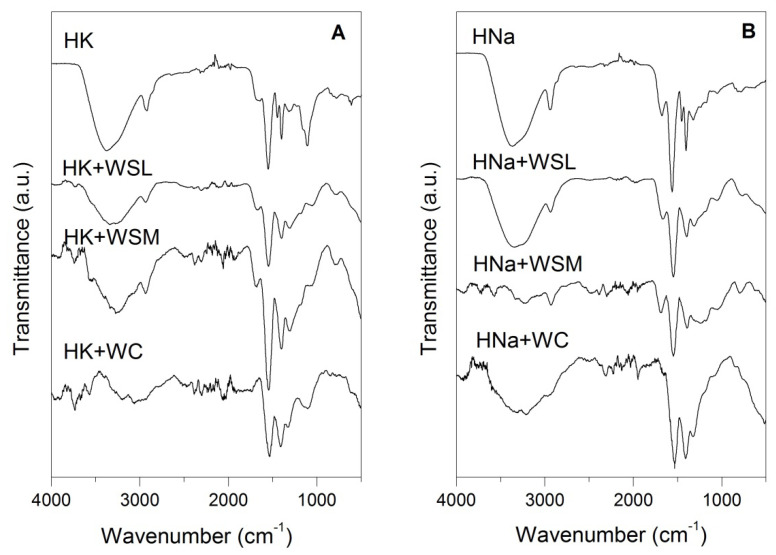
FTIR spectra of hydrogels before and after the swelling process. (**A**) HK and (**B**) HNa.

Figure 4 shows the FTIR spectra of commercial hydrogels before and after the swelling process. In the case of hydrogels without exposure to the swelling process, the characteristic bands indicated in Table 2 were identified. When comparing the spectra before and after the swelling process, insignificant changes were observed when the hydrogels were exposed to WSL; however, when hydrogels exposed to WSM, WC and WSN references undergo significant changes in intensity, wavenumber and morphology in the bands of the functional groups of OH (~3370), CH2 (~2937 and ~2860), C-OH (~1674), COO- (~1555 and ~1450), CC (~1162), and other deformations in other functional groups in bands close to ~815, 784, ~616, and ~489. These modifications suggest changes in the chemical structures of hydrogels, such as ion exchange or breaking of crosslinkings, that justify the decrease in the swelling performance in the hydrogels. One of the most evident transitions after exposure to different aqueous phases is present in the band of deformation of the OH bond manifested at ~3370, indicating the transformation of and/or decrease in this type of bond in the polymer. This change can be caused by the breakage and loss of crosslinking, as well as the decrease in the hydrophilic capacity of the polymer when the polar side groups of the chain are altered, reducing the presence of the OH bond.

### 3.5. Physicochemical Analysis

**Figure 5:**
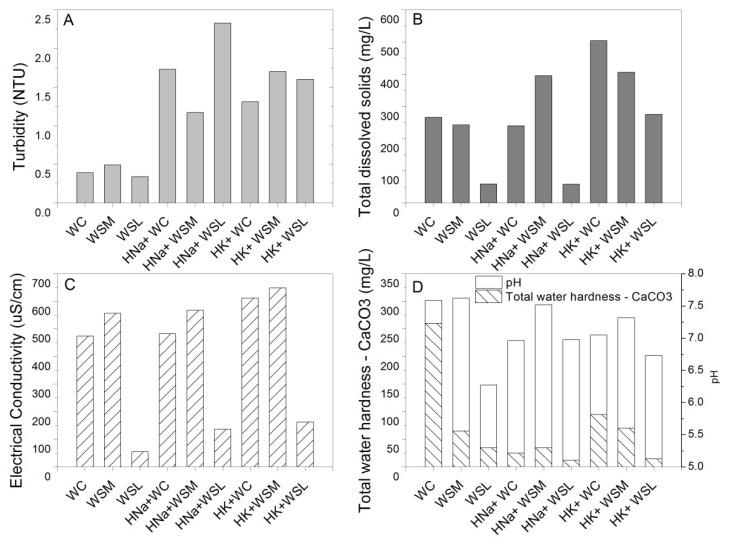
Physical properties comparison of the types of waters before and after the swelling process. (**A**) Turbidity. (**B**) Total dissolved solids. (**C**) Electrical conductivity. (**D**) Total water hardness—pH.

**Figure 6:**
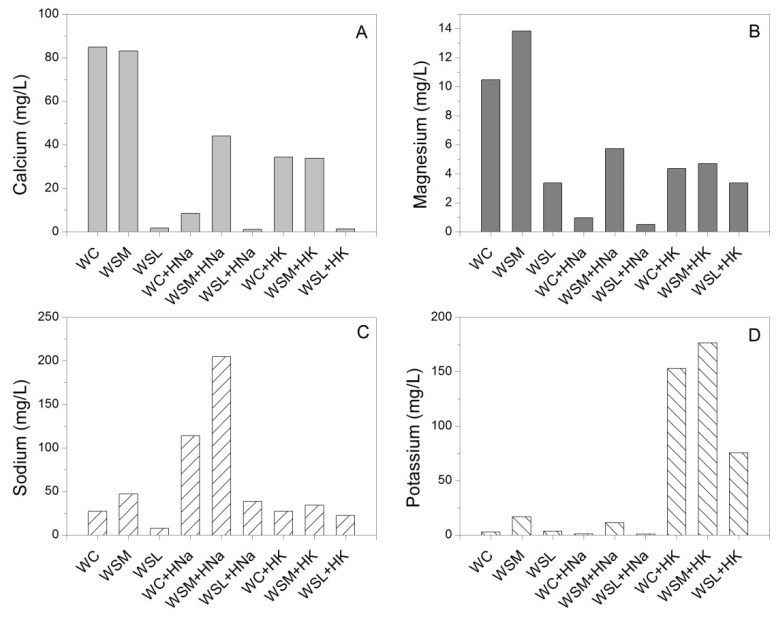
Cations comparison of the types of waters before and after swelling process. (**A**) Calcium, (**B**) Magnesium, (**C**) Sodium, and (**D**) Potassium.

Figure 5 shows some physical properties of the different types of waters recovered after the swelling process. Note that turbidity has significant changes, increasing after the swelling process, indicating the possible release of substrates by the hydrogels in all aqueous media studied (Figure 5A). In this same context, changes are observed concerning the amount of total dissolved solids in several water samples, especially in the types of waters that were used in the swelling processes of potassium-based hydrogels (Figure 5B). The electrical conductivity remains constant in almost all samples except for the waters where the potassium-based hydrogel swelling process was carried out, which have slight increments as well as the sodium-based hydrogel with the WSL swelling process (Figure 5C). Regarding the water hardness, it is observed that the WC reference has high contents of CaCO_3_; this may be one of the reasons related to the degradation of hydrogels as observed in the swelling test (items 3.1, 3.2, and 3.3). In this context, calcium cations can interact more strongly with the ionic fraction (potassium or sodium) belonging to the polymeric chain of the hydrogel, decreasing its polar character, thus decreasing the swelling and retention capacity of these superabsorbent materials, in this context, this characteristic justifies the results of the water in terms of reduction in hardness and calcium ions (Figure 5D) and on the other hand explains the increase in sodium and potassium in the water (Figure 6C,D). Moreover, the pH shows slight changes that can be explained by the exchange in ions between the water and the polymer.

The contents of some metals (calcium, magnesium, sodium, and potassium) common in the types of waters are shown in Figure 6 before and after the swelling process. The results associated with the presence of these metals suggest the possibility of ionic exchange between different types of waters and hydrogels in a way that sodium and potassium contained in the hydrogel structure are exchanged by calcium and magnesium ions contained in commercial waters, previous work has shown this interchange for different types of hydrogels [15,18]. In this sense, the results of this test suggest that the calcium and magnesium cations of the aqueous phase are exchanged by the ionic fractions of sodium and potassium of the polymeric phase during the swelling process. It is noteworthy that this ion exchange phenomenon does not favor swelling according to the kinetic curves observed in Figure 1, Figure 2 and Figure 3.

### 3.6. Dynamic Light Scattering (DLS) and Zeta-Potential

**Figure 7:**
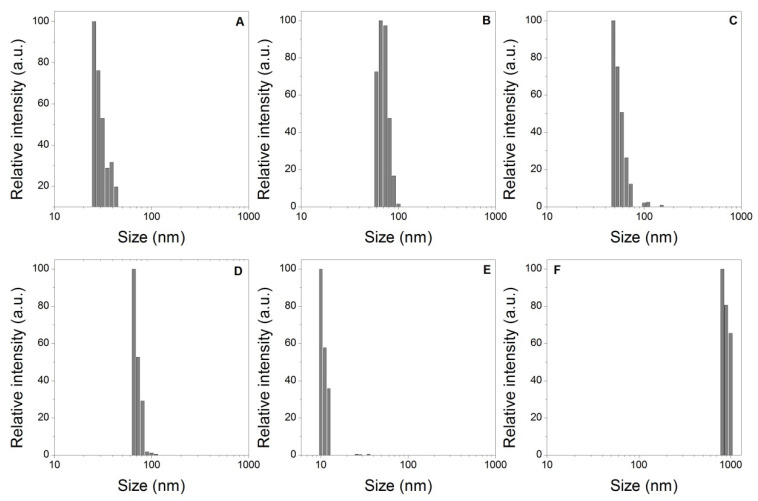
Dynamic light scattering particle size distribution of wastewaters: (**A**) WC + HNa. (**B**) WSM+ HNa. (**C**) WSL + HNa. (**D**) WC + HK. (**E**) WSM+ HK. (**F**) WSL + HK.

The presence of particles, most of them nanometric scale, was found in the wastewaters released during the swelling process (see Figure 7), it is noteworthy that all the types of waters were examined before the swelling process and it was not possible to verify the presence of nanoparticles. Likewise, it was not possible to verify the presence of nanoparticles in the distilled water after of swelling process. In this context, the presence of these particles in wastewater from the swelling process suggests the detachment of particles from the three-dimensional structure of the hydrogel, these nanoparticles are polymeric chains of low molecular weight with fragile crosslinking bridges that are broken and consequently released into water.

**Figure 8:**
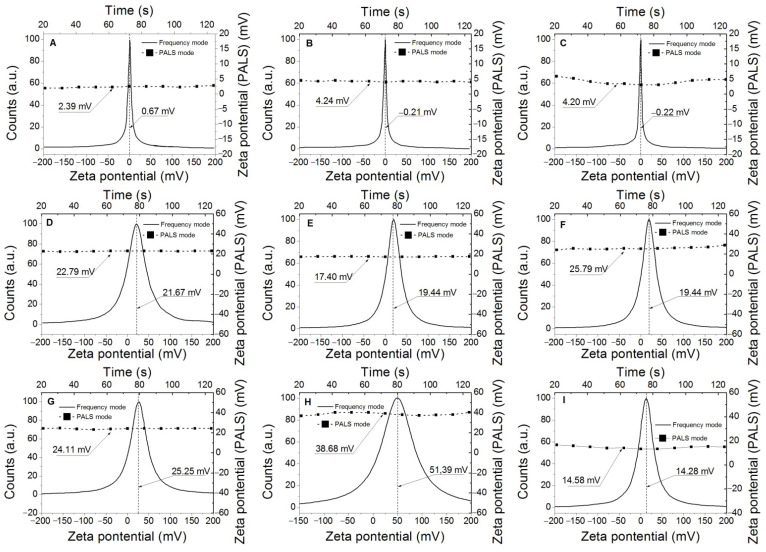
Zeta potential distribution of pure and wastewater: (**A**) WC. (**B**) WSM. (**C**) WSL. (**D**) WC + HNa. (**E**) WSM+ HNa. (**F**) WSL + HNa. (**G**) WC + HK. (**H**) WSM+ HK. (**I**) WSL + HK.

Figure 8 presents the results related to the Zeta-Potential of the types of wastewaters showing the charge present in the wastewater obtained by the Phase Analysis Light Scattering (PALS) and frequency analysis modes. In the case of commercial waters samples (WC, WSM, and WSL), relatively neutral charges are observed, close to zero; these results agree with the impossibility of detecting colloids by DLS in the same samples. On the other hand, as expected, the wastewater (WC + HNa, WSM + HNa, WSL + HNa, WC + HK, WSM + HK, and WSL + HK) show cationic charges in both Zeta-Potential techniques used, charges associated with nanoparticles that are generated by the detachment of low molecular weight polymer chains. These low molecular weight polymer chains (nanoparticles) are weakly linked to the polymer network by crosslinkings that are broken by the ionic attack of different types of water.

**Figure 9:**
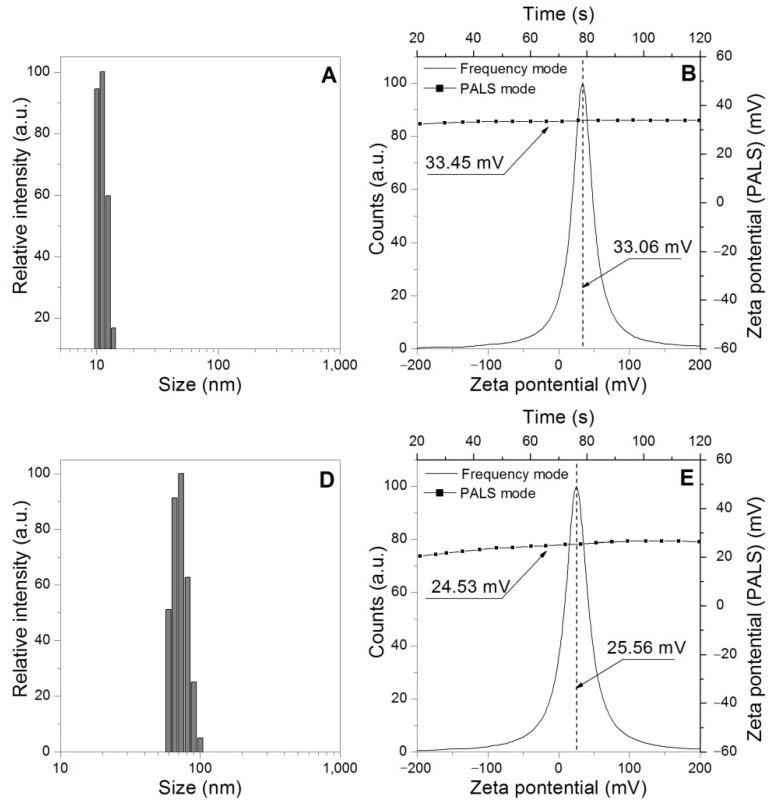
Dynamic light scattering particle size distribution and Zeta potential distribution of Ca(NO_3_)_2_ solution residue after of swelling process. (**A**) DLS of Ca(NO_3_)_2_+ HK. (**B**) Zeta potential of Ca(NO_3_)_2_+ HK. (**D**) DLS of Ca(NO_3_)_2_+ HNa. (**E**) Zeta potential of Ca(NO_3_)_2_+ HNa.

To verify the described phenomena, the DLS and Zeta-Potential techniques were applied to the residual solution of Ca(NO_3_)_2_, obtained after the swelling process of the two commercial hydrogels, see Figure 9. The results show the presence of suspended colloids in both residual solutions; even so, the cationic charges associated with the suspended particles can be observed, confirming the release of nanometric polymer particles from the hydrogels.

### 3.7. UV-vis Analysis

**Figure 10:**
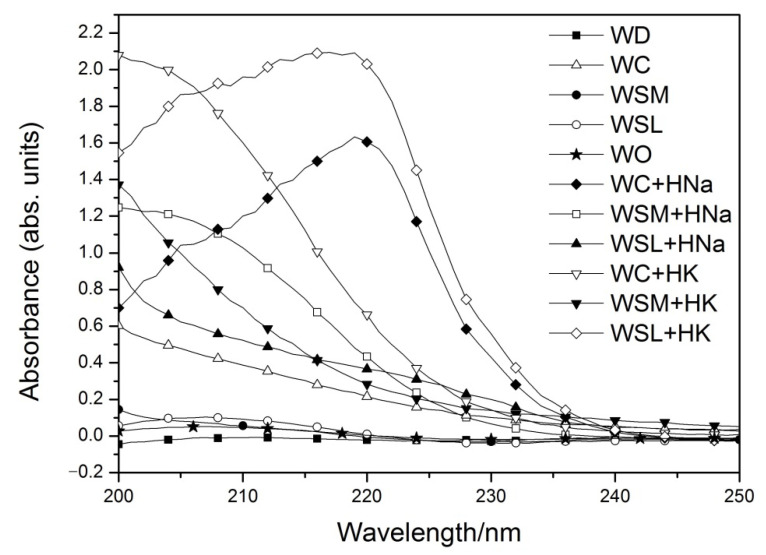
UV-vis spectra of pure waters and wastewaters.

Figure 10 shows the UV-vis spectra of the different water samples studied. It is observed that the water samples before the swelling process (WD, WC, WSM, WSL, and WO) have detectable minimum absorbances that change significantly after the swelling process. This change in absorbance is due to the presence of released substrates (ions, salts, and/or polymeric nanoparticles) in the water.

**Figure 11:**
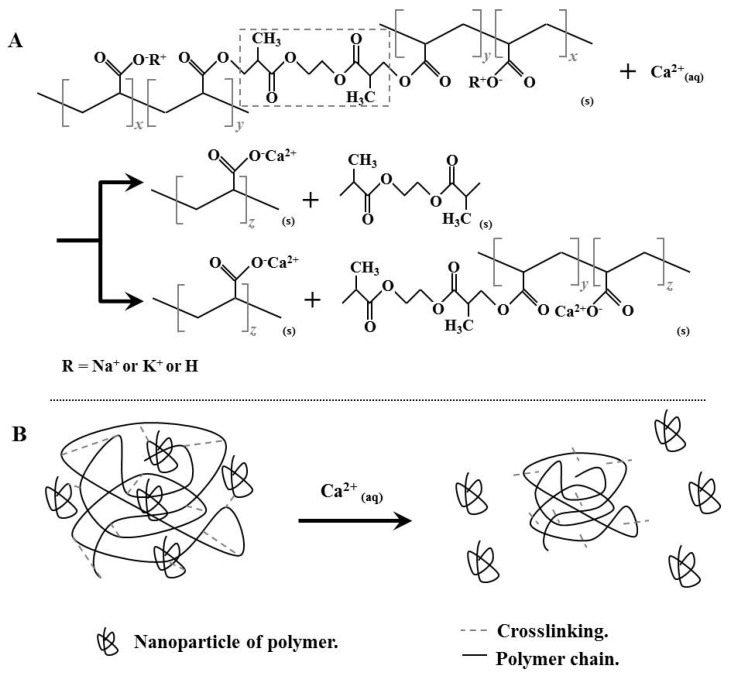
Schematic representation of the effects of solutions associated with calcium ions in commercial hydrogels. (**A**) Schematic representation of the degradation of the polymers, (**B**) Schematic representation of the generation of nanoparticles by crosslinking breaking.

The results obtained suggest several effects on the hydrogel structural network caused by the presence of some ions present in water such as calcium, in addition to a possible synergistic effect of degradation due to the joint effect of several types of ions (Ca^2+^, Mg^2+^, K^+^, Na^+^, etc.); on the other hand, physicochemical changes are observed in some types of waters. One of the effects found is the breaking or cleaving of the crosslinking bridges that connect the polymer chains, which consequently causes the release of low molecular weight polymer chains identified as nanoparticles by DLS and Zeta-potential; said nanoparticles are finally released in the solutions or wastewater after the swelling process and remain suspended after the structural collapse of the hydrogel. In addition, the ion exchange of metals present in the side polar groups of the polymeric chain of the hydrogel by other types of ions present in water and prepared solutions stand out, changing both the efficiency and functionality of the hydrogels as well as the contents of ions associated with potassium, sodium, calcium, and magnesium in commercial waters and prepared solutions, see Figure 6. In Figure 11, a schematic representation suggesting the effects observed in the interaction between solutions with different types of ions and hydrogels can be seen regarding ion exchange and crosslinking breakdown. The degradation observed in the hydrogels can be explained in the two ways suggested in Figure 11A; likewise, said degradation allows inferring the release of nanoparticles (Figure 11B).

## 4. Conclusions

The polymeric degradation of hydrogels based on potassium and sodium polyacrylate was studied as a result of the interaction with substrates present in absorbable fluids by this type of polymer. Likewise, this interaction between the liquid and the polymer generates transformations in both phases in physical-chemical aspects. In this context, ions present in the different types of waters or other types of solutions such as Ca^2+^, Mg^2+^ or Na^+^ can cleavage the crosslinkings and change the ionic fraction of the polymeric chain of the hydrogels, reducing the retention capacity and/or the useful life of this type of superabsorbent polymer. These changes in the three-dimensional network of the studied hydrogels lead to the structural collapse of this type of polymer, leading to the fractioning of the network and, consequently, the release of particles of low dimensional scales.

On the other hand, during the swelling process, the types of waters or chemical solutions in contact with the hydrogels are altered in several physicochemical characteristics caused by breaks of crosslinking and ionic exchange between the aqueous phase and the polymeric phase; this phenomenon has to be considered for several processes where it is essential not to generate cross-contamination in the aqueous medium surrounding the hydrogels at the time of swelling. Finally, the phenomena described in this article can be considered favorable from the point of view of ionic exchange between the studied phases; however, it can be unfavorable from a cross-contamination point of view. This is why the authors invite the development of hydrogels from renewable sources, such as biomass, that can be integrated in an environmentally friendly way.

## Figures and Tables

**Table 1:** Main characteristics of the commercial hydrogels.

	Potassium Polyacrylate (HK)	Sodium Polyacrylate (HNa)
Generic chemical formula	[-CH_2_-CH(COOK)-]n	[-CH_2_-CH(COONa)-]n
Purity (%)	~96	~95
Molecular weight by GPC (Mw)	~4000	~5100
Particle size (Mesh)	20–40	5–10
Crosslinker	Ethylene glycol dimethacrylate (EGDMA)	Ethylene glycol dimethacrylate (EGDMA)
Crosslinking density * (Croslinking unit per Monomer units)	950–1350	700–1100

* Relationship used for the synthesis of hydrogels.

**Table 2:** Characteristic Bands of hydrogels based on potassium and sodium polyacrylate.

	Description	Deformation	Wavenumber (cm^−1^)
**HK**	Stretching vibration of the hydroxyl group.	O-H	~3369
	Asymmetric and Symmetric stretch.	CH_2_	~ 2935 & ~2860
	Deformation vibrations.	C-OH	~ 1674
	Asymmetric and symmetric stretching and another associated deformation of the group.	COO^-^	~ 1555, ~1451, ~1404, ~1317 & ~1169
	Stretching vibrations of C-O bond and deformation vibrations of C-O-H group.	C-O & C-O-H	~1239
	Bond deformation.	C–C	1162
	Bond stretching in the carboxyl acid structure.	C=O	~1113
	Other characteristic deformations of polymeric hydrogel based on potassium.	--	~855, ~820, ~784~638 & ~616
**HNa**	Stretching vibration of the hydroxyl group.	O-H	~3383
	Asymmetric and Symmetric stretch.	CH_2_	~ 2930 & ~2865
	Deformation vibrations.	C-OH	~1659
	Asymmetric and symmetric stretching and another associated deformation of the group.	COO^-^	~ 1555, ~1451, ~1404, ~1322 & ~1162
	Stretching vibrations of C-O bond and deformation vibrations of C-O-H group.	C-O & C-O-H	−1235
	Bond deformation.	C–C	1162
	Bond stretching in the carboxyl acid structure.	C=O	~1128
	Other characteristic deformations of hydrogel based on sodium.	--	~1047, ~815, ~774 & ~621

**Table 3:** Samples of water and residual water from the swelling process.

References Sample	Type of Sample	References Sample	Type of Sample
HK	Hydrogel based on potassium	HNa	Hydrogel based on sodium
WD	Distilled Water	HK + WD	Hydrogel after swelling process
WO	Osmosis Water	HK + WO	
WC	Commercial water 1	HK + WC	
WSM	Commercial water 2	HK + WSM	
WSL	Commercial water 3	HK + WSL	
WSN	Water of supply net	HNa + WD	
CaCl_2_+ HK	Saline solutions with distilled water after of swelling process	HNa + WO	
MgCl + HK		HNa + WC	
KCl + HK		HNa + WSM	
NaCl + HK		HNa + WSL	
CaCl_2_ + HNa	Saline solutions with distilled water after of swelling process	WD + HK	Wastewater after the swelling process
MgCl + HNa		WO + HK	
KCl + HNa		WC + HK	
NaCl + HNa		WSM+ HK	
Ca + HK	Calcium-associated solutions with distilled water after of swelling process	WSL + HK	
Ca(NO_3_)_2_ + HK		WD + HNa	
CaCO_3_ + HK		WO+ HNa	
Ca + HNa	Calcium-associated solutions with distilled water after of swelling process	WC + HNa	
Ca(NO_3_)_2_ + HNa		WSM+ HNa	
CaCO_3_ + HNa		WSL + HNa	

**Table 4:** Physicochemical properties of the types of waters.

Water Reference	Turbidity	Total Dissolved Solids	Total Water Hardness CaCO_3_	pH	Electrical Conductivity	Anions				Cations			
						Chlorides (Cl^-^)	Sulfates (SO_4_^−2^)	Nitrates (NO_3_^-^)	Nitrites (NO_2_^-^)	Calcium	Magnesium	Sodium	Potassium
	NTU	mg/L	mg/L	--	uS/cm	mg/L	mg/L	mg/L	mg/L	mg/L	mg/L	mg/L	mg/L
WC	0.39	266	260	7.56	478.34	35.09	177.71	8.8	0.96	84.86 *	2.69	10.49	27.1
WSM	0.49	242	65	7.62	553.67	59.88	142.55	1.3	0.28	83.14	16.79 *	13.84 *	47.01 *
WSL	0.34	59	35	6.27	54,33	29.99	12.12	1.43	0.31	1.66	3.58	3.36	7.55
WSN	1.85	1880	378.33	7.09	793.4	51.98	276.08	51.85	0.01	79.95	9.84	37.71	4.07

* The concentration used for the formulation of saline and calcium solutions.
